# RNA-Seq Analysis of Spinal Cord Tissues from hPFN1^G118V^ Transgenic Mouse Model of ALS at Pre-symptomatic and End-Stages of Disease

**DOI:** 10.1038/s41598-018-31132-y

**Published:** 2018-09-13

**Authors:** Caroline Barham, Daniel Fil, Stephanie D. Byrum, Yasir Rahmatallah, Galina Glazko, Mahmoud Kiaei

**Affiliations:** 1 Department of Pharmacology and Toxicology, University of Arkansas for Medical Sciences, Little Rock, AR USA; 2 Department of Biochemistry and Molecular Biology, University of Arkansas for Medical Sciences, Little Rock, AR USA; 3 Department of Geriatrics, University of Arkansas for Medical Sciences, Little Rock, AR USA; 4 Department of Neurology, University of Arkansas for Medical Sciences, Little Rock, AR USA; 5 Department of Biomedical Informatics, University of Arkansas for Medical Sciences, Little Rock, AR USA; 6 Present Address: Department of Biochemistry and Molecular Genetics, University of Alabama at Birmingham, Birmingham, AL USA

## Abstract

Amyotrophic lateral sclerosis (ALS) is a fatal neurodegenerative disease that leads to the loss of motor neurons. The molecular mechanisms of motor neuron degeneration are largely unknown and there are currently no effective therapies to treat this disease. In this work, we report whole transcriptome profiling of spinal cords of mutant transgenic hPFN1^G118V^ mice and their wildtype transgenic hPFN1^WT^ controls at a pre-symptomatic stage and at the end-stage of disease. Analyses revealed that end-stage hPFN1^G118V^ mice had 890 differentially expressed genes (747 up-regulated, 143 down-regulated) when compared to pre-symptomatic hPFN1^G118V^ mice, and they had 836 differentially expressed genes (742 up-regulated, 94 down-regulated) when compared to age-matched hPFN1^WT^ controls. Pre-symptomatic hPFN1^G118V^ mice were not significantly different from age-matched hPFN1^WT^ controls. Ingenuity Pathway Analysis identified inflammatory pathways significantly activated in end-stage hPFN1^G118V^ samples, suggesting an excess of glial activation at end-stage disease, possibly due to an increase in glial composition within the spinal cord during disease progression. In conclusion, our RNA-Seq data identified molecules and pathways involved in the mechanisms of neurodegeneration that could potentially serve as therapeutic targets for ALS.

## Introduction

Amyotrophic lateral sclerosis (ALS) is a fatal neurodegenerative disease characterized by the loss of upper and lower motor neurons. Individuals affected by the disease develop progressive muscle weakness and atrophy, eventually leading to death due to respiratory failure^1,2^. While clinical studies and basic research have provided insight into mechanisms of ALS, no causative and treatable mechanism has been identified.

For more than 10 years, the complex pathogenesis of ALS has been evaluated with a variety of gene expression profiling methods, such as microarrays and RNA sequencing (RNA-Seq), coupled with whole-tissue or laser-capture microdissected tissue at several stages of disease in mutant SOD1 ALS mouse models^3–8^ and in postmortem patient tissues^9–14^. Most studies that analyzed gene expression in ALS mouse models (reviewed in^15^) were conducted before RNA-Seq had been developed or made widely accessible, so they relied heavily on microarray techniques. While useful and relatively inexpensive, microarray experiments often are limited in the number of genes that can be evaluated (sometimes less than 2,000 genes), which limits the detection scope to transcripts corresponding to genomic sequencing data that is available in the public domain at the time the experiments are conducted^16^. RNA-Seq, however, has the advantage of using virtually all RNAs and corresponding cDNA sequences in the tissue, which enables detection of virtually all known and novel transcripts in the cells or tissues. Additionally, background noise is lower with RNA-Seq than with microarrays, and RNA-Seq bypasses technical issues inherent to microarrays, such as cross-hybridization, nonspecific hybridization, and limited dynamic range^16,17^.

Recently, our lab developed transgenic mouse lines that overexpress human profilin1. One strain carries the gene with a mutation at position 118 (hPFN1^G118V^), and the other carries a wild-type copy (hPFN1^WT^)^18^. A second mouse model with mutation in PFN1 that over expresses PFN1^C71G^ has been reported with robust ALS-like symptoms and pathologies^19^. hPFN1^G118V^ is one of eight identified profilin1 mutations that have been reported in ALS patients^20,21^. The hPFN1^G118V^ mouse model exhibits many key clinical and pathological signs consistent with human ALS, including loss of lower and upper motor neurons, aggregation of mutant profilin1, activation of glial cells, fragmented mitochondria, muscle atrophy, weight loss, abnormally ubiquitinated proteins, reduced expression of choline acetyltransferase, and reduced survival^18^. We examined transcriptomic changes in spinal cord tissue of hPFN1 mice to gain insights into the mechanism(s) of mutant hPFN1 neurotoxicity. Unlike human transcriptomic analyses, which are limited to tissues from patients with end-stage ALS, this mouse model provides the opportunity to examine changes that occur pre-symptomatically in the central nervous system, in addition to those that occur at the end-stage of disease.

The aim of this work was to identify molecular changes in spinal cords of the hPFN1^G118V^ ALS mouse model at pre-symptomatic and end-stages. To our knowledge, this is the first study to use next-generation RNA-Seq to measure gene expression in hPFN1^G118V^ mice at pre-symptomatic and end-stages. We report evidence that the overall transcriptome profiles of spinal cord tissues were highly similar, and that those of hPFN1^G118V^ mice with end-stage disease clustered away from those of hPFN1^WT^ mice. This study led to the discovery of 890 genes that were differentially expressed in mutant mice with end-stage disease, as compared to mutant mice that were pre-symptomatic (i.e., 50 days old); of the 890 genes, 747 were up-regulated and 143 were down-regulated.

## Results

### RNA-Seq data analysis

Sixteen spinal cord samples from male hPFN1^G118V^ and age-matched hPFN1^WT^ mice were used in this study. The experiments used male hPFN1^G118V^ mice that were pre-symptomatic (50 days old; mutant young [MY]) or at the end-stage of disease (175–245 days old; mutant old [MO]), as well as age-matched hPFN1^WT^ mice (wild-type young [WY], and wild-type old [WO], respectively) (Table 1). When hPFN1^G118V^ mice could not right themselves within 20 seconds, they were considered at end-stage of disease^18^. Female mice were excluded to reduce effects on gene expression due to hormonal fluctuations of the estrus cycle. The numbers of sequenced fragments before and after the cleaning step, the lengths of cleaned sequences, and the GC content of cleaned sequences are presented in Table 1. After gene expression levels were calculated and unexpressed genes filtered out, 18,167 genes remained.

**Table 1:** Sample identity and RNA-Seq statistics.

Mouse ID	Genotype^1^	Age (days)	Batch	Total sequenced fragments	Total cleaned fragments	Sequence length	%GC	qRT-PCR^2^
MO1	MUT	198	1	31595682	30955215	36–76	48	X
MO2	MUT	190	1	37449858	36869443	36–76	48	X
MO3	MUT	245	1	51360499	50499947	36–76	48	X
MO4	MUT	224	2	63553644	62373141	36–76	49	
MO5	MUT	175	2	47682463	47120118	36–76	49	
MY1	MUT	50	1	44323436	43616751	36–76	48	X
MY2	MUT	50	1	42110571	41432780	36–76	48	X
MY3	MUT	50	1	37757883	37093439	36–76	48	X
WO1	WT	190	1	49828561	48951584	36–76	48	X
WO2	WT	190	1	39015596	38404019	36–76	48	X
WO3	WT	190	1	35855593	35207083	36–76	48	X
WO4	WT	294	2	72735989	71927022	36–76	50	
WO5	WT	294	2	65738082	64949263	36–76	49	
WY1	WT	50	1	34559215	34048886	36–76	47	X
WY2	WT	50	1	46519005	45747075	36–76	48	X
WY3	WT	50	1	45006658	44309399	36–76	48	X

^1^MUT, hPFN1^G118V^; WT, hPFN1^WT^.

^2^“X” indicates samples assayed for qRT-PCR.

**Figure 1:**
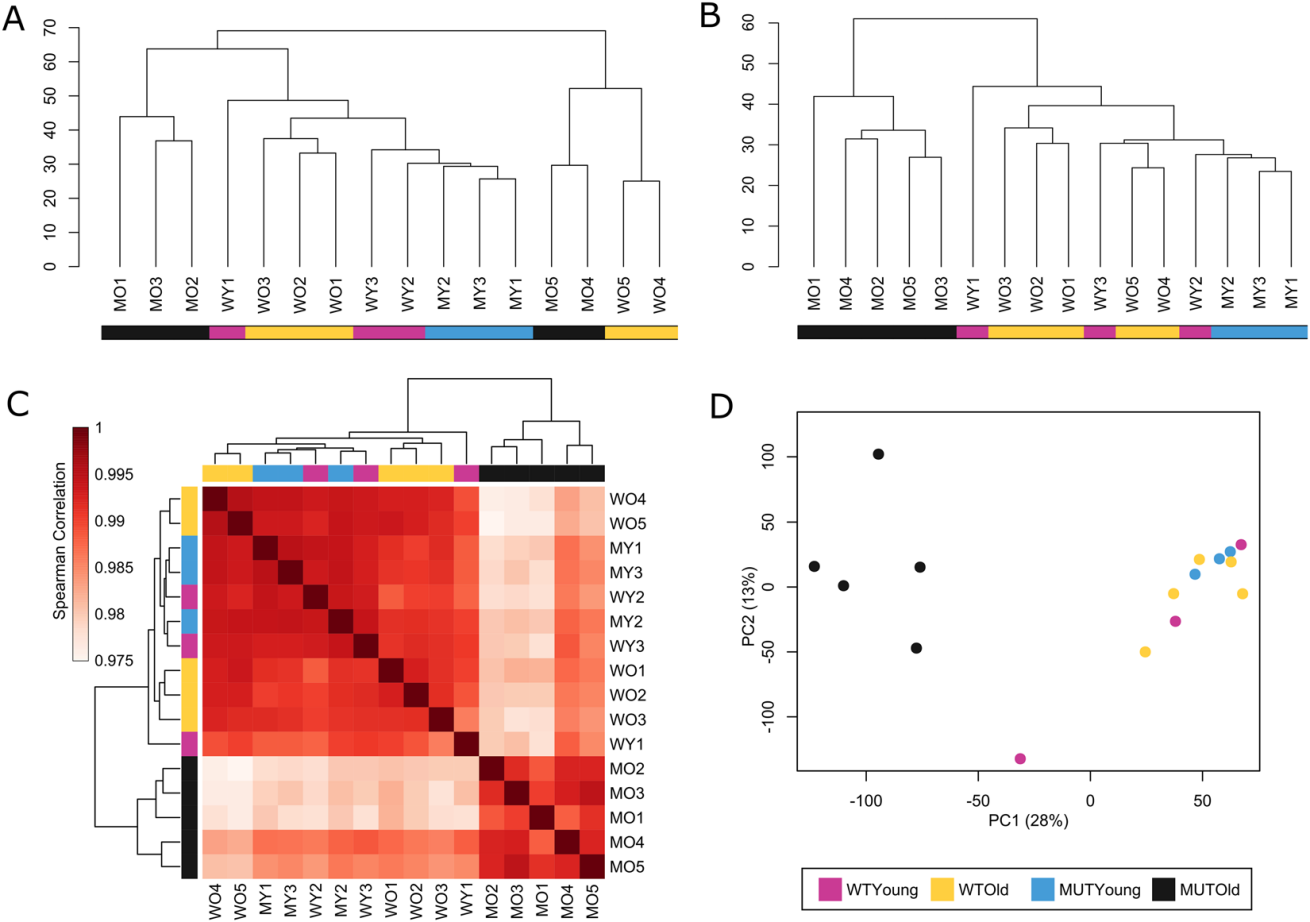
Analysis of 16 spinal cord samples based on normalized expression values of 18,167 genes. (**A**,**B**) Unsupervised hierarchical clustering. (**A**) Separation of batch 1 and batch 2; no batch-effect correction. (**B**) Post-batch-effect correction; batch separation minimized, and clearly separate clustering of MO samples from the other samples. (**C**) Heatmap showing the pair-wise Spearman’s rank correlation between all samples and clustering of MO samples apart from the other three groups. (**D**) PCA plot of samples in which MO mice are clustered away from the other three groups.

The sequence data analysis of spinal cord RNA samples in 2 batches showed significantly fewer gene sequences were detected in batch 1 than in batch 2, suggesting a potential batch effect between the two libraries. Unsupervised hierarchical clustering of the filtered log_2_(1 + FPKM) data before batch correction revealed that samples from batch 1 and 2 clustered into two separate clusters, independent of age and genotype (Fig. 1A). After batch-effect correction, unsupervised hierarchical clustering showed a clear separation of end-stage mutant mice from the other three groups (WO, WY, and MY) (Fig. 1B). The clustering of MO samples from different batches reveals biological reproducibility within the MO samples and illustrates the importance of the batch-correction step. Spearman’s rank correlation coefficient clustering (Fig. 1C) of the batch-effect-corrected data revealed similar separation of MO from all other samples. However, it should be noted that the lowest value of all Spearman’s rank correlation comparisons in this data set was 0.975, indicating that even the most different spinal cord transcriptomes were highly correlated. Principle component analysis (PCA) confirmed clustering of MO samples apart from all others, in agreement with Spearman’s and unsupervised hierarchical clustering analysis (Fig. 1D). No recognizable clustering pattern from WO, WY, and MY samples was detected in unsupervised hierarchical clustering, Spearman’s rank correlation, or PCA, suggesting a high degree of similarity among the three groups.

### Gene expression specificity by cell type

**Figure 2:**
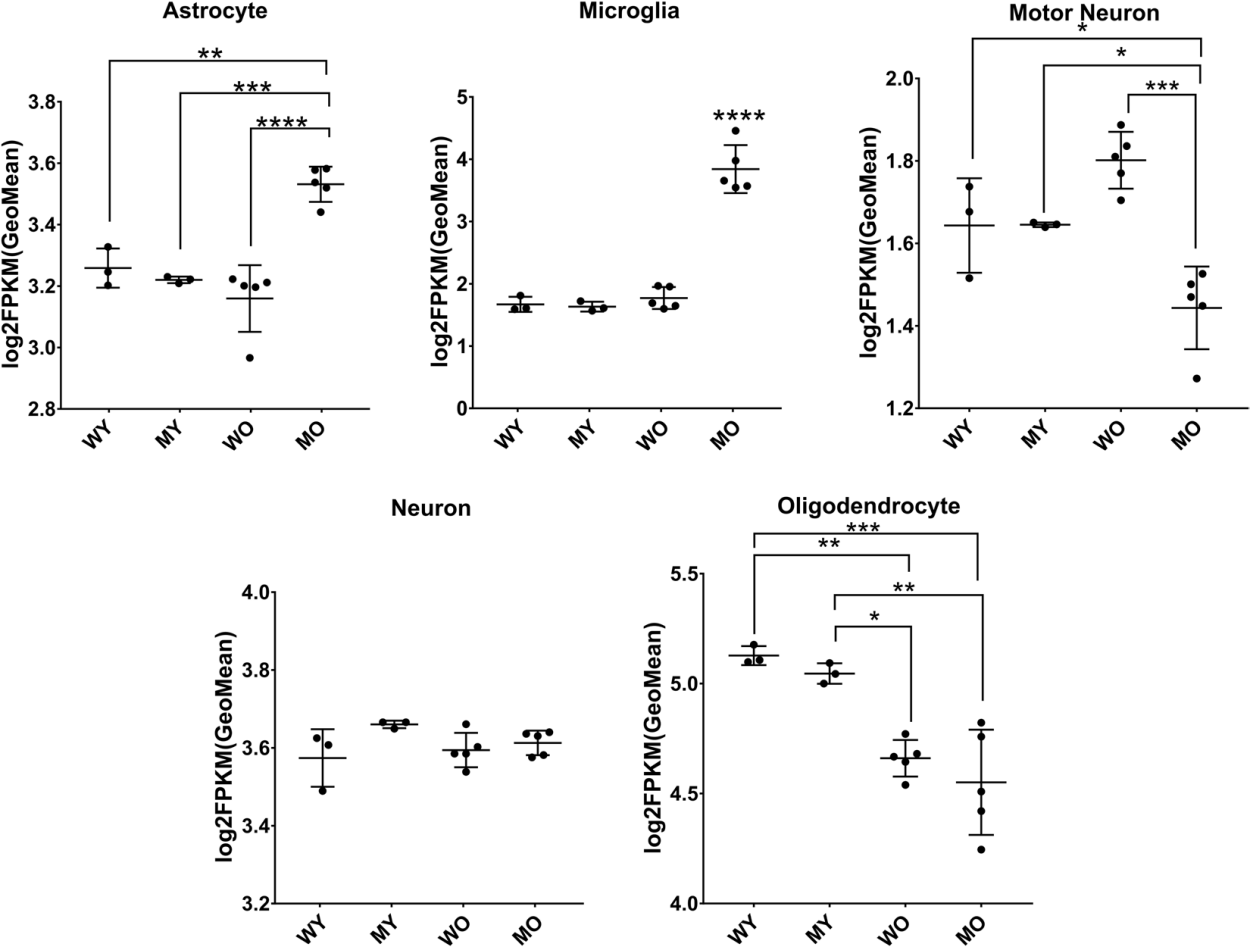
Cell-type composition of spinal cord samples. Changes in specific cell types indicated by geometric mean of the log_2_(1 + FPKM) values of cell-type marker genes for each cell type. Statistical differences in cell type composition are calculated by the Tukey Kramer test. Number of marker genes used per cell type: 60 for astrocyte, 5 for microglia, 5 for motor neuron, 72 for neuron, and 35 for oligodendrocyte. Error bars indicate SD; *P < 0.05, **P < 0.01, ***P < 0.001, ****P < 0.0001.

We verified the cell-type compositions of the spinal cord samples by analyzing expression of genes specifically expressed by neurons, motor neurons, astrocytes, oligodendrocytes, and microglia (Fig. 2, genes are listed in Supplementary Table S1)^10,22^. In MO samples, astrocyte and microglia compositions were significantly higher and motor neuron composition was significantly lower than in WO, WY, and MY. Significant changes in oligodendrocytes appeared to be dependent on the age of the mice in WO and MO samples, compositions of oligodendrocytes were significantly lower than in each of the younger samples but were not statistically different from each other. Neuron populations were not significantly different among the four groups.

### Differential expression

**Figure 3:**
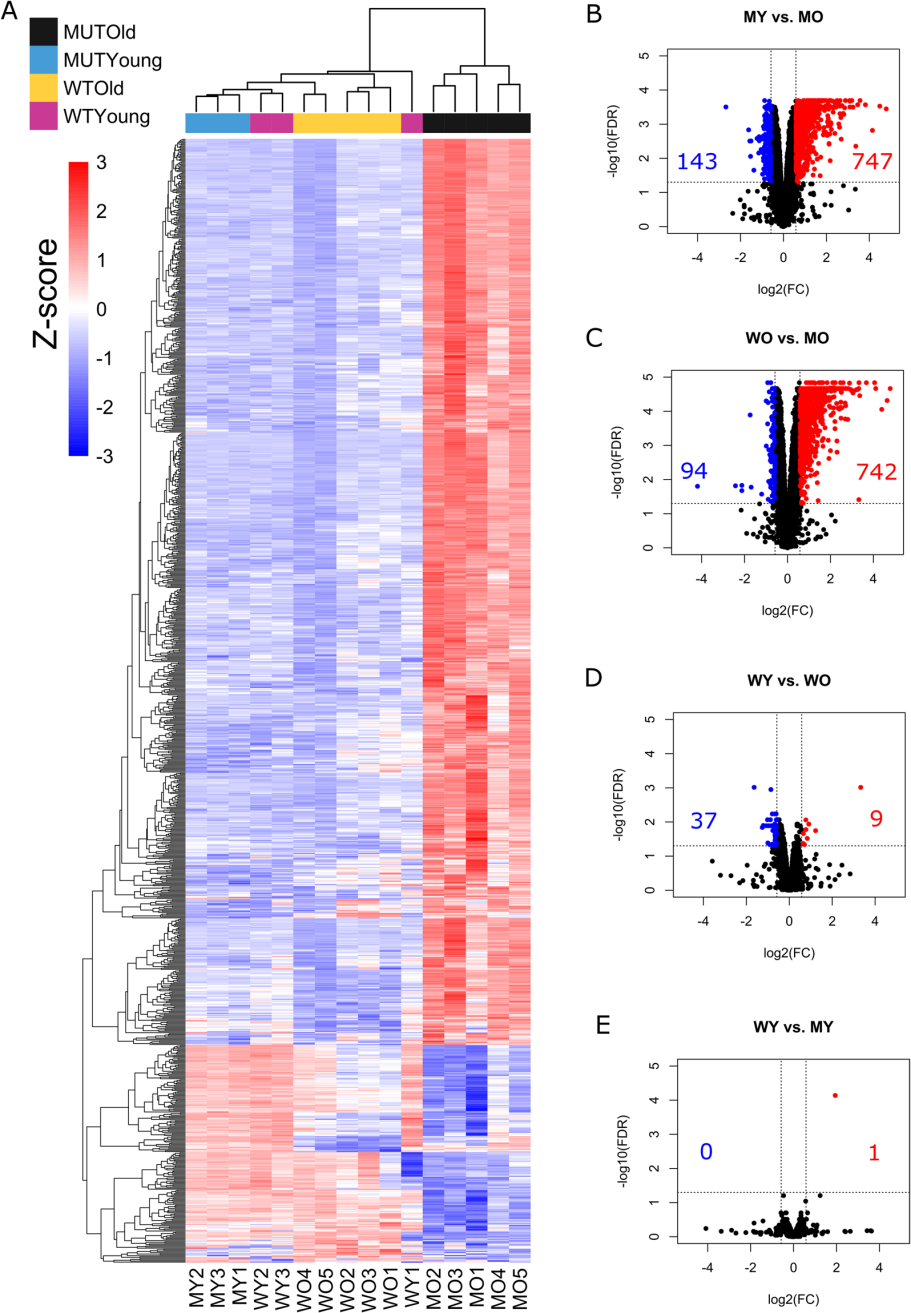
Heatmap and volcano plots of differential expression. (**A**) Heatmap shows 1,048 significantly (FDR ≤ 0.05) differentially expressed genes across four comparisons (MY vs. MO, WO vs. MO, MY vs. WY, WY vs. WO) from each spinal cord sample. Colors above the heatmap indicate mouse age and genotype. Each row of the heatmap represents the z-score transformed log_2_(1 + FPKM) values of one differentially expressed gene across all samples (blue, low expression; red, high expression). (**B**–**E**) Volcano plots of significantly differentially expressed genes, (FDR ≤ 0.05 and |FC| ≥ 1.5; red, up-regulated; blue, down-regulated). Numbers of genes up-regulated or down-regulated are denoted.

To determine the differential gene expression between presymtomatic and end-stage hPFN1^G118V^ mice and age-matched hPFN1^WT^ mice, we used *limma*, an open-source R-based software package that analyzes differential expression of log_2_-transformed FPKM values^23^. A total of 1,048 genes were significantly (FDR < 0.05) differentially expressed with an absolute fold change of 1.5 or greater for four comparisons: WO vs. MO, MY vs. MO, WY vs. WO, and WY vs. MY (Supplementary Table S2). A heatmap shows that most differentially expressed genes were up-regulated in spinal cord samples from the MO group (i.e., hPFN1^G118V^ mice at end-stage; Fig. 3A).

**Figure 4:**
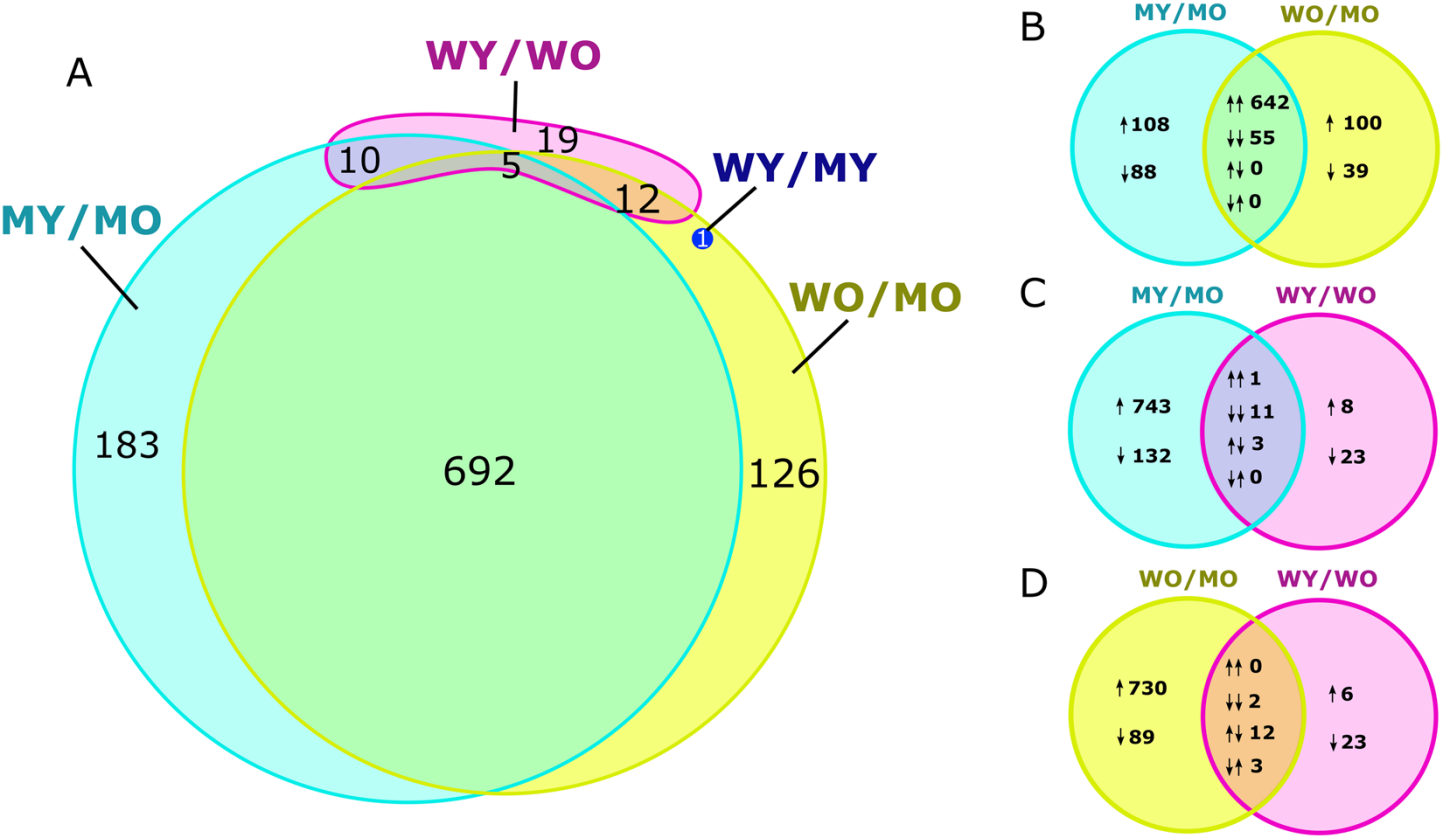
Venn diagrams of differential expression. (**A**) Venn diagram indicating the number of significantly differentially expressed genes (FDR ≤ 0.05 and |FC| ≥ 1.5) across the four comparisons (MY vs MO, WO vs MO, WY vs WO, WY vs MY). (**B**–**D**) Detailed analysis between three comparisons illustrating overlap between up- and down-regulated genes. Pairs of arrows in intersection refer to direction of fold change in comparisons on the left and right sides, respectively.

Expression varied most between hPFN1^G118V^ mice that were at end-stage of disease and those that were pre-symptomatic (MY vs. MO), in which 890 genes were differentially expressed, with 747 up-regulated and 143 down-regulated, as shown in the volcano plot (Fig. 3B). When end-stage hPFN1^G118V^ mice were compared to age-matched hPFN1^WT^ mice (WO vs. MO), 836 differentially expressed genes were identified, with 742 up-regulated and 94 down-regulated (Fig. 3C). A subset of 697 genes were differentially expressed in both MO vs. MY and WO vs. MO comparisons as shown by Venn diagram (Fig. 4A); of these, 642 were up-regulated, 55 were down-regulated, and none were regulated in opposite directions in both comparisons (Fig. 4B). The WY vs. WO comparison revealed that only 46 genes are differentially expressed (9 up-regulated, 37 down-regulated) (Fig. 3D); 27 of the 46 were shared with the MY vs. MO comparison, the WO vs. MO comparison, or both, suggesting potential overlap of dys-regulated expression of specific genes involved in progression of motor neuron disease and aging (Fig. 4A). Analysis of WY vs. MY identified only one significantly dys-regulated gene, *Prnp*, which was up-regulated (Fig. 3E). This gene was significantly up-regulated also in the WO vs. MO comparison (Fig. 4A).

### Pathway analysis

**Figure 5:**
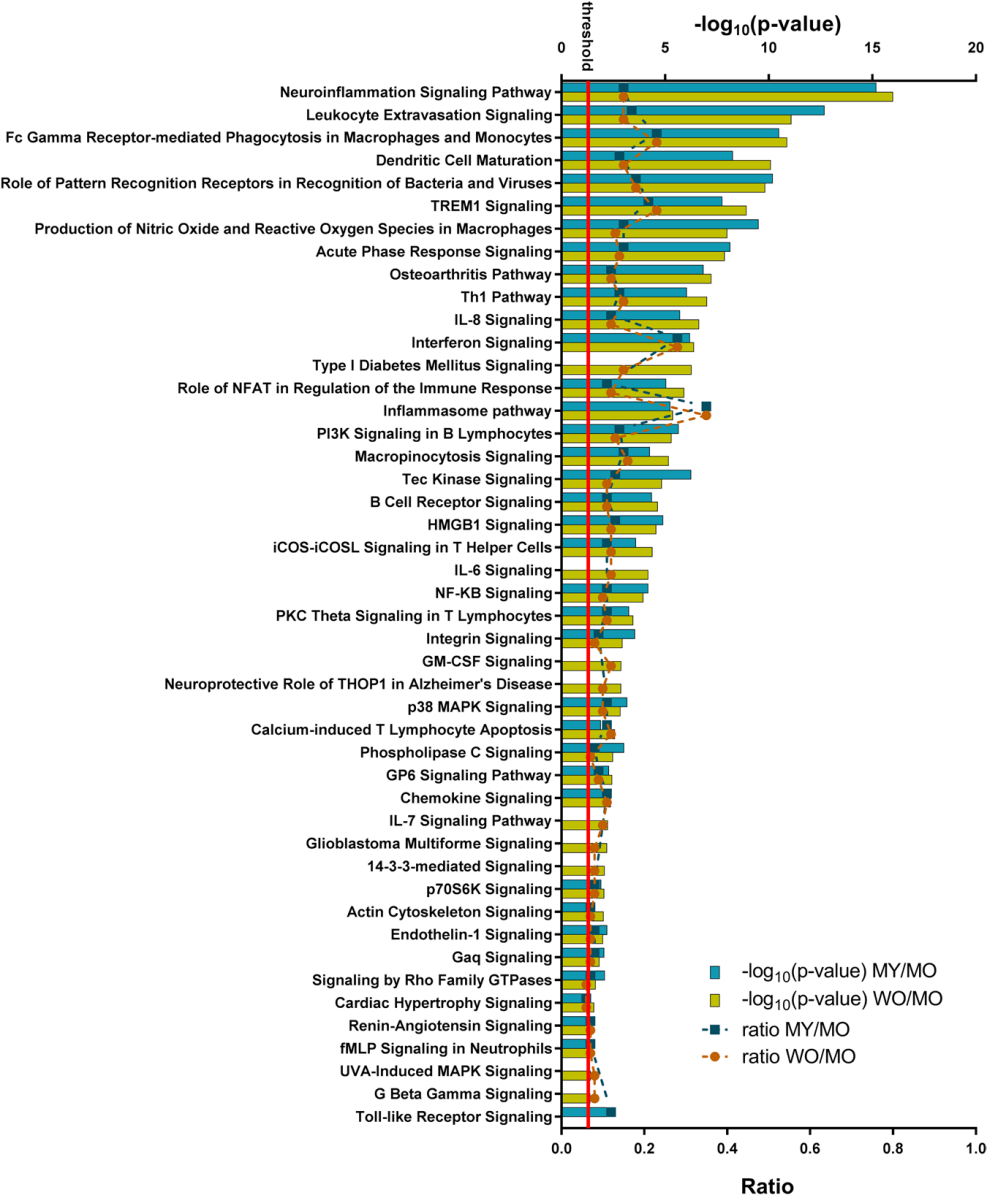
Significantly activated pathways in hPFN1^G118V^ spinal cord tissue. IPA identified pathways (P ≤ 0.05) that are significantly activated (z-score ≥2) in differentially expressed genes from MY vs. MO and/or WO vs. MO comparisons. The –log_10_(P-value) is reported on the upper axis, with the threshold P-value indicated by a red line at –log_10_(P-value) = 1.3. The ratio between the number of differentially expressed genes and the number of genes in the specific pathway is reported on the bottom axis. Lack of a bar from MY vs. MO or WO vs. MO for a specific pathway indicates that the comparison did not meet the criteria of P ≤ 0.05 and/or z-score ≥2.

Ingenuity Pathway Analysis identified functionally relevant cellular pathways that were linked to differentially expressed genes in the MY vs. MO (890 genes) and WO vs. MO (836 genes) comparisons. Because the ideal set size for IPA from gene expression data is 200–3,000, we could not analyze the 46-gene set from the WY vs. WO comparison. Of the identified pathways that were considered significant (Fisher Exact P-value < 0.05; Supplementary Table S3), IPA predicted significant activation (z-score >2) of 37 pathways in the MY vs. MO comparison and 45 pathways in the WO vs. MO comparison (Fig. 5).

**Figure 6:**
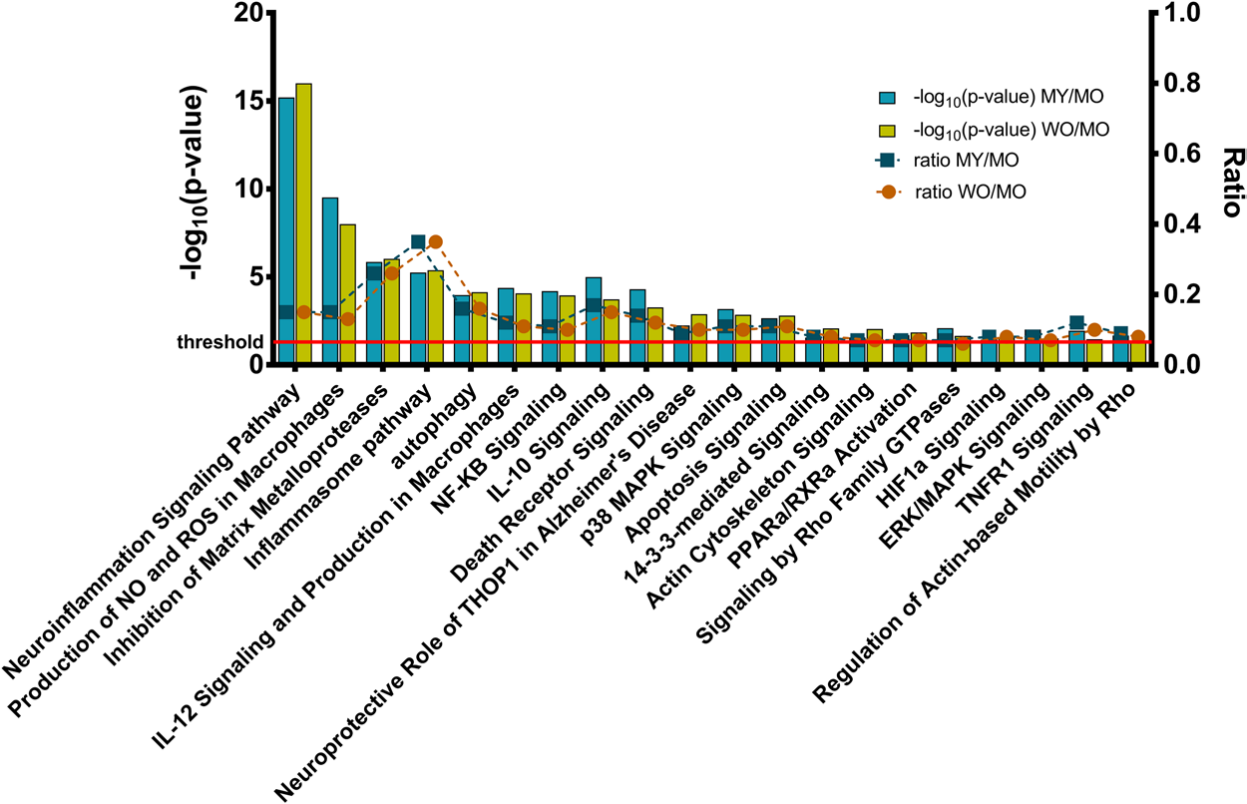
ALS-relevant pathways. IPA identified significantly activated pathways that are relevant to ALS. The –log_10_(P-value) is reported on the left axis, with the threshold P-value (P ≤ 0.05) indicated by a red line at –log_10_(P-value) = 1.3. The ratio between the number of differentially expressed genes and the number of genes in the specific pathway is reported on the right axis. (NO, nitric oxide; ROS reactive oxygen species).

Pathways with biological relevance to ALS are listed in Fig. 6. Differentially expressed genes corresponded to, most notably, components of neuroinflammation signaling, radical-generating pathways, inhibition of matrix metalloproteases, inflammasome pathway, autophagy, interleukin signaling and production, NF-kB signaling, apoptosis, MAP kinase signaling, and actin cytoskeleton signaling. The neuroinflammation signaling pathway had one of the lowest P-values of the identified pathways in both comparisons and was predicted to be significantly up-regulated (z-score of ~5.6 for WO vs. MO and ~5.5 for MY vs. MO). Of the genes that were linked to selected ALS-relevant pathways, the highest degree of overlap occurred with those linked to the inflammasome. End-stage hPFN1^G118V^ samples (i.e., MO) had significantly increased levels of transcripts encoding components of inflammasome complexes (*Pycard*, *Aim2*, *Casp1*, *Naip2*), as well as other molecules important to the inflammasome pathway (*Casp8*, *Tlr4*, *Il1b*, *Ctsb*).

### Validation of RNA-Seq results

RNA-Seq results were validated with qRT-PCR analysis of RNA samples from spinal cords of mice from batch 1. From the differentially expressed genes identified in the four comparisons, we randomly selected 12 candidates with |log_2_FC| > 0.58 (or |FC| > 1.5) and log_2_(1 + FPKM) > 1 across all samples for the given gene (Table 2).The rationale for selecting genes at random for validation was to avoid scientific bias. Because *Prnp* was the only significantly differentially expressed gene in the WY vs. MY comparison, it was included among the 12 genes in the validation set. At least one up-regulated gene and one down-regulated gene was chosen from the genes identified in the other three comparisons for validation of RNA-Seq.

**Table 2:** List of qRT-PCR validated genes*.

Gene Symbol	Description	MY/MO	WO/MO	WY/MY	WY/WO
Abca8a	ATP-binding cassette, sub-family A (ABC1), member 8a	+			+
Ccl6	chemokine (C-C motif) ligand 6	+	+		
Chodl	chondrolectin	−	−		
Fmod	fibromodulin	+	+		
Hmgcs1	3-hydroxy-3-methylglutaryl-Coenzyme A synthase 1	−			−
Opalin	oligodendrocytic myelin paranodal and inner loop protein				−
Prnp	prion protein		+	+	
S100a4	S100 calcium binding protein A4	+	+		
Serpina3n	serine (or cysteine) peptidase inhibitor, clade A, member 3 N	+	+		
Tlr2	toll-like receptor 2	+	+		
Trem2	triggering receptor expressed on myeloid cells 2	+	+		
Vim	vimentin	+	+		

*Comparisons in which genes were found to have |log_2_FC | ≥ 0.58 (or |FC| ≥ 1.5) are denoted by + for up-regulation and – for down-regulation.

**Figure 7:**
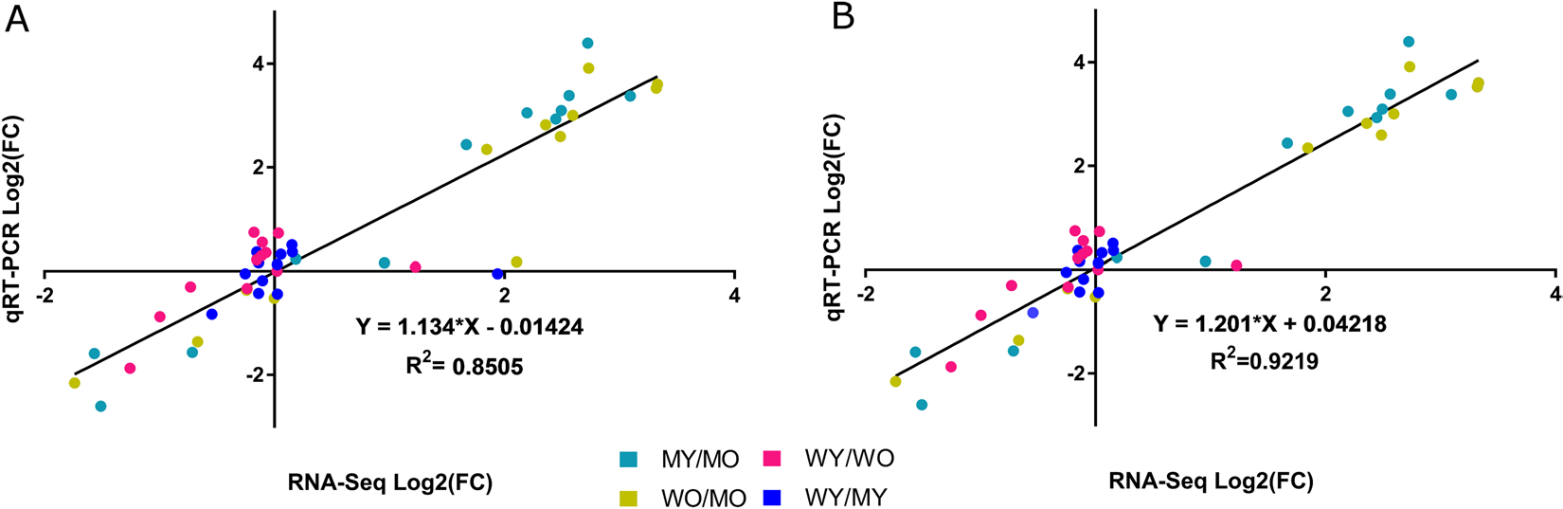
Results from qRT-PCR validation. Linear regression plots between log_2_(FC) values computed by *limma* from RNA-Seq data and log_2_(FC) values detected by qRT-PCR for 12 selected genes across four comparisons: MY vs. MO, WO vs. MO, WY vs. WO, and WY vs. MY. (**A**) Linear regression plot of all samples measured. (**B**) Linear regression plot of all samples except *Prnp* qRT-PCR outliers from WO vs. MO and WY vs. MY comparisons.

Results of the qRT-PCR analysis confirmed those of RNA-Seq for all genes across all four comparisons, with the exception of *Prnp* expression in WO vs. MO and in WY vs. MY. When comparing WO and MO, RNA-Seq analysis of *Prnp* resulted in log_2_(FC) of 2.10, but qRT-PCR resulted in log_2_(FC) of 0.182. When comparing WY and MY, RNA-Seq analysis of *Prnp* resulted in log_2_(FC) of 1.94, but qRT-PCR resulted in log_2_(FC) of −0.048. The RNA-Seq data showed that *Prnp* was up-regulated when hPFN1^G118V^ mice were compared to hPFN1^WT^ mice within the same age groups, but the qRT-PCR data showed only insignificant changes in log2(FC) of *Prnp*. When all RNA-Seq-derived log_2_(FC) values for the set of 12 genes in all four comparisons were correlated with the qRT-PCR log_2_(FC) values, the values were highly consistent (Fig. 7A). However, when *Prnp* results from WO vs. MO and from WY vs. MY were removed from the analysis, the Pearson correlation coefficient increased from R = 0.92 (P < 10^−15^) to R = 0.96 (P < 10^−15^), and the coefficient of determination increased from R^2^ = 0.85 to R^2^ = 0.92 (Fig. 7B).

## Discussion

ALS is a complex neurodegenerative disease with no viable treatment, and its causative mechanistic pathways have yet to be elucidated. In this study, we used RNA-Seq to evaluate transcriptomic changes in spinal cord tissues of hPFN1^G118V^ mice as compared to those of age-matched hPFN1^WT^ mice, at pre-symptomatic (50 days; the earliest time at which hPFN1 aggregation has been shown to occur in hPFN1^G118V^ mice) and end (~202 days) stages of disease.^18^ This RNA-Seq data analysis is an initial step in determining the transcriptional changes caused by mutant PFN1 and may be further confirmed by comparing/contrasting RNA-Seq data from PFN1^C71G^ mouse model.^19^

Overall, transcriptomes of the spinal cord tissues were highly similar across the different groups investigated—the lowest Spearman’s correlation was 0.975. However, normalized gene expression clearly distinguished the end-stage hPFN1^G118V^ mice from the other three groups, as shown by hierarchical clustering and PCA analyses.

It has been proposed that astrocytes and microglia play important roles in the pathogenesis and progression of ALS^24,25^, and, consistent with this, our results indicated increased prevalence of astrocytes and microglia in end-stage hPFN1^G118V^ mice. It is likely that changes in the heterogeneous cell populations of the spinal cord at end-stage, especially increases in glial population and activity, can be attributed to this observation. This is in agreement with findings in our recent report on the hPFN1 mouse model, in which increased astrocytic and microglial activation were revealed by immunohistochemical analysis of the ventral horn of end-stage hPFN1^G118V^ mice^18^. It would be of great interest to address the role of astrocytes and microglia in the PFN1^C71G^ mouse model in the near future.

The high degree of upregulation of the neuroinflammation signaling pathway in spinal cord tissues of end-stage PFN1^G118V^ mice could be related to the observed increase in astrocytes and microglia in this group. Many inflammation-related pathways were up-regulated, including those of NF-kB signaling, production of nitric oxide and reactive oxygen species, interleukin signaling, and the inflammasome. Previous transcriptomic studies of murine models and of post-mortem human ALS tissues also identified upregulation of inflammatory processes^9,10,13,26,27^. Inflammasomes are multiprotein complexes that are activated by a variety of factors and induce activation of caspase-1, which mediates production of pro-inflammatory cytokines interleukin-1B and interleukin-18, inducers of pyropoptosis, a highly inflammatory form of programmed cell death^28^. Genes encoding caspase-1 and apoptosis-associated speck-like protein containing a CARD (ASC, also known as PYCARD), which are components of all types of inflammasome complexes, were significantly up-regulated in end-stage PFN1^G118V^ spinal cords^29^. AIM2 and NAIP2 transcripts, which are present in AIM2 inflammasomes and NLRC4 inflammasomes, respectively, also were up-regulated in tissues of PFN1^G118V^ mice at end-stage^29^. The NLRP3 inflammasome complex has been characterized most extensively and implicated in ALS and other neurodegenerative diseases that include Alzheimer’s disease, Parkinson’s disease, frontotemporal dementia, and Huntington’s disease^28,30–32^. Investigations of human ALS tissue samples and SOD1 mouse models have shown up-regulation of NLRP3 inflammasome activity^31,32^. Although transcripts encoding the NLRP3 protein were not significantly up-regulated in end-stage hPFN1^G118V^ mice, it is possible that AIM2, NLRC4, and, potentially, NLRP3 inflammasomes play a role in PFN1-mediated death of motor neurons. The ways in which PFN1 mutation causes increased inflammation and possible up-regulation of inflammasomes in spinal cords of our mouse model may be worth examining in the future as a potential therapeutic target.

We selected 50 days as the time point to evaluate the pre-symptomatic stage in our mouse model because this is the earliest age at which PFN1 has been found in the insoluble fractions of spinal cords of these mice^18^. Only one gene, *Prnp*, was significantly differentially expressed at the pre-symptomatic stage in hPFN1^G118V^ mice, as compared to age-matched hPFN1^WT^ mice. Significant upregulation of *Prnp* was identified by RNA-Seq comparisons of hPFN1^G118V^ and hPFN1^WT^ mice at both time points, revealing that this gene could be expressed at significantly greater levels in mutant mice at early ages, before disease onset. However, qRT-PCR analyses did not reveal *Prnp* up-regulation in these two comparisons, and the correlation between RNA-Seq and qRT-PCR data improved when data from *Prnp* in these comparisons was removed from validation experiments. Both hPFN1^G118V^ and hPFN1^WT^ mice were generated with a plasmid vector (i.e., MoPrP.Xho1) that contains the mouse *Prnp* promotor^18,33^. In addition to the mouse *Prnp* promotor, the vector contains 5′ intronic and 3′ untranslated sequences, with an Xho1 cleavage site between exons 2 and 3 of *Prnp*^33^. It is possible that the alignment software could have aligned the endogenous *Prnp* gene to the intronic and untranslated sequences of the *Prnp* gene from mRNA generated by the transgenic expression vector. If the hPFN1^G118V^ mice transcribe significantly more transgene than the hPFN1^WT^ mice, misalignment of transgenic mRNA sequences as endogenous *Prnp* mRNA could be the cause of the up-regulation of *Prnp* in hPFN1^G118V^ mice that was observed with RNA-Seq but not with qRT-PCR. Lack of any other significantly differentially expressed genes in the WY vs. MY comparison suggests that, at a transcriptomic level, 50-day-old hPFN1^G118V^ mice are hardly distinguishable from 50-day-old hPFN1^WT^ mice.

Given these findings, a future study is needed to examine gene expression changes at multiple time points after 50 days and before end-stage. Evaluation of spinal cord gene expression at more points in the disease, such as at early onset and full onset should be considered. Limiting gene expression studies to end-stage evaluation may indicate mainly molecular events that are secondary to causative mechanisms. Evaluating time points that occur soon after preliminary disease onset in this mouse model would also help identify molecular pathology of ALS disease progression and pathways that could be targeted therapeutically. Additionally, more time points would enhance detection of molecular changes that occur before and during the process of motor neuron death, as opposed to those that occur after motor neuron death (i.e., at end-stage).

It is possible that changes in the heterogeneous cell population of the spinal cord, especially increases in numbers and activation of glial cells that are observed at the end-stage of the disease, can blur molecular changes that occur in other cell types that are relevant to ALS, such as motor neurons. For instance, doubling the population of astrocytes may double expression levels of a given gene expressed by astrocytes, so expression changes evaluated by whole spinal cord transcriptome sequencing may not be due to increases of expression within individual cells. In future studies of hPFN1 mice, it would be worth exploring changes in gene expression with cell-type-specific RNA isolation methods that have been used with other ALS disease models. This would further elucidate whether the results we have presented are related to increased glial cell composition of the spinal cord, or due to other secondary effects. Methods developed to isolate microglia from spinal cord tissue have been used in RNA-Seq analysis of the SOD1 G93A ALS mouse model^34^. Additionally, embryonic stem-cell differentiation methods have been used to analyze expression profiles of astrocytes and their interplay with motor neurons in the SOD1 G93A mouse model^35^. In the RNA-Seq study by Phatnani *et al*., RNA sequencing data from both whole spinal cord tissues and from neurons and astrocytes derived from embryonic stem cells were analyzed in tandem to provide dynamic insight into the contributions of glial cells in ALS^35^. Previous transcriptome studies in both mouse models and humans have been conducted on laser microdissected motor neurons, enhancing detection of molecular changes in the neurons without bias from other cell types^5,6,11^. Use of any of these methods with the hPFN1 mouse model could add more dimension to the transcriptional effects of mutant PFN1 throughout disease progression.

This is the first study to profile the gene expression of hPFN1^G118V^ mice, and it provides a foundation for future gene expression profiling studies of this ALS mouse model. The data presented here from whole spinal cord tissue could be used together with future studies that acutely isolate cells of the central nervous system or that examine specific cells differentiated from embryonic stems. Our findings of transcripts encoding components of AIM2 and NLRC4 inflammasomes causes us to infer that inflammasomes are promising targets to be studied in the mouse models for PFN1-mediated death of motor neurons. These molecules in the inflammasome complexes may form the basis of future studies to lead the identification of molecular features that could potentially be therapeutic targets for ALS.

## Materials and Methods

### Mice and tissue collection

The experiments used male hPFN1^G118V^ mice that were pre-symptomatic (50 days old; mutant young [MY]) or at the end-stage of disease (175–245 days old; mutant old [MO]), as well as age-matched hPFN1^WT^ mice (wild-type young [WY], and wild-type old [WO], respectively). These transgenic mice were developed with a vector construct in which hPFN1^G118V^ or hPFN1^WT^ cDNA was inserted at the Xho1 site of the MoPrP.Xho expression plasmid^18,33^. Detailed information regarding the identity of spinal cord samples (including age, genotype, and ID number) used for RNA-Seq and real-time quantitative reverse transcription (qRT-PCR) can be found in Table 1. Mice were housed under 12-hour light/dark conditions and were fed 4–5 g chow (Harlan/Teklad #7001) per day per mouse, with free access to water. Mouse identification was performed by genotyping^18^. Animals were sacrificed and spinal cords collected at either 50 days or at end-stage of disease for subsequent molecular analyses. hPFN1^G118V^ mice were considered at end-stage of disease when they could not right themselves within 20 seconds^18^. In this study we used animals and all the experimental procedures that involved mice for this study were approved by UAMS Institutional Animal Care and Use Committee (IACUC), AUP protocol # 3730 and conducted in full accordance of the guidelines of the Institutional Animal Care and Use Committee at the University of Arkansas for Medical Sciences. The Animal Care and Use Program at the University of Arkansas for Medical Sciences has an Assurance with the NIH (#A3063-01/D16-00035), is USDA registered (71-R-011), and AAALAC International accredited (000322). The animal facilities are located within the Division of Laboratory Animal Medicine (DLAM) in three distinct areas on campus. The animal care and use program is centrally managed by DLAM administration and is physically housed in approximately 30,000 square feet of space. DLAM has surgical, procedural, and housing facilities which focus on several research interests on campus. There are three full time veterinarians, as well as administrative staff and AALAS certified husbandry technicians to support all animal based research on this campus.

### RNA library construction and sequencing

Total RNA was isolated with the TRIzol Reagent protocol (ThermoFisher, Waltham, MA) from spinal cords of hPFN1^WT^ and hPFN1^G118V^ mice. RNA quality and concentration were estimated with Bioanalyzer 2100 and RNA 6000 Nano Kit (Agilent Technologies, Waldbronn, Germany). RNA-Seq libraries were prepared with Illumina TruSeq Stranded mRNA LT Sample Preparation Kit (Illumina, San Diego, CA) using 1 μg of total RNA according to the manufacturer’s protocol. Sequencing was performed on the NextSeq. 500 platform (Illumina). RNA-Seq data used for downstream analysis in this study came from two separate batches of RNA libraries (denoted as batch 1 or batch 2 in Table 1).

### Processing RNA-Seq data

FastQC software (https://www.bioinformatics.babraham.ac.uk/projects/fastqc/) was used to generate quality-control reports of individual FASTQ files, before and after filtering. Trimmomatic^36^ was used to filter out low-quality reads and possible contamination from Illumina Adaptors or PCR primers. The remaining reads were aligned with TopHat^37^ to the UCSC mm10 mouse reference genome. Cufflinks^37^ was used to quantify gene expression in fragments per kb of transcript per million mapped read (FPKM). This normalization accounts for differences in library size and gene length.

Prior to batch-effect removal, the FPKM matrix of all 16 samples was log_2_-transformed, after adding 1 to avoid undefined values. Unexpressed genes were filtered out by removing genes with 0 expression in at least 8 samples; 18,167 genes in 16 samples survived the filtering criterion. Because samples were prepared in two batches, function ComBat^38^ from Bioconductor package *sva*^39^ was then used to remove possible batch effects between batches 1 and 2 by applying parametric empirical Bayesian adjustments to mean and scale. All downstream data analyses were performed on log_2_-transformed FPKM values after correction for batch effects.

### Differential gene expression and pathway analysis

Gene expression was analyzed with the *limma* package, an R-based open-source software designed to analyze transcriptomic data for differential expression^23^. Differentially expressed genes were identified by applying a false discovery rate (FDR) cut-off of 0.05 and an absolute fold-change (FC) of 1.5. To identify significant canonical pathways in which differentially expressed genes were enriched, pathway enrichment analysis was conducted with Ingenuity Pathway Analysis software (IPA; Ingenuity Systems, Redwood City, CA).

### Cell composition analysis

Changes in cell-type compositions of spinal cord samples were estimated according to expression of cell-type-specific genes for microglia, astrocytes, oligodendrocytes, neurons, and motor neurons^10,22^. The genes were identified from a published list of human genes^10^; therefore, we selected those with corresponding mouse orthologues (Supplementary Table S1). We calculated geometric means by using log_2_(1 + FPKM) values instead of FPKM values.

### qRT-PCR validation

RNA samples from batch 1 were used for qRT-PCR validation. cDNA was synthesized from DNase-treated total RNA (1 μg) with High Capacity cDNA Reverse Transcription kit according to the manufacturer’s instructions (Applied Biosystems, Foster City, CA). qRT-PCR was performed with primers from IDT and SYBR Green PCR Master Mix (Applied Biosystems), according to the manufacturer’s instructions, and detected with the ABI QuantStudio 12 K Flex platform (Invitrogen, Carlsbad, CA). Reactions were carried out in duplicate with 52 ng of cDNA and 250 nM of each primer in a final volume reaction of 20 μl (50 °C for 2 minutes, 95 °C for 2 minutes; 45 amplification cycles at 95 °C for 15 s, 60 °C for 1 min). Relative expression levels were determined by the comparative threshold cycle (ΔΔCt) method, using GAPDH as a reference gene.

## Electronic supplementary material

Supplementary Info

Dataset 1

Dataset 2

Dataset 3

## Data Availability

The data set generated and analyzed during this study is available in the GEO repository with accession number GSE113924.
