# Supplementary material for: RNA-Seq Analysis of Spinal Cord Tissues from hPFN1G118V Transgenic Mouse Model of ALS at Pre-symptomatic and End-Stages of Disease
Source: Sci Rep. 2018 Sep 13;8:13737. doi: 10.1038/s41598-018-31132-y (PMC6137178; doi:10.1038/s41598-018-31132-y)
Supplement: Supplementary file 1 — Supplementary Info [file 41598_2018_31132_MOESM1_ESM.pdf]

Supplementary Material

RNA-Seq Analysis of Spinal Cord Tissues from hPFN1^{G118V} Transgenic Mouse Model of ALS at Presymptomatic and End Stages of Disease

Caroline Barham[1], Daniel Fil[1,Ψ], Stephanie D. Byrum[2], Yasir Rahmatallah[5], Galina Glazko[5], and Mahmoud Kiaei[1,3,4,*]

[1]Department of Pharmacology and Toxicology, [2]Department of Biochemistry and Molecular Biology, [3]Department of Geriatrics, [4]Department of Neurology, [5]Department of Biomedical Informatics, University of Arkansas for Medical Sciences, Little Rock, AR, USA

Legends for Supplementary Tables

Supplementary Table S1. Cell-type composition genes. List of cell-type-specific genes for astrocytes, microglia neurons, motor neurons, and oligodendrocytes (collected from D'Erchia et al.) that were used for cell-type composition analysis of spinal cord samples. We selected only genes with corresponding mouse orthologues showing $\log_2(1+FPKM) > 0$ in all samples.

Supplementary Table S2. Significantly differentially expressed genes from limma. Four lists of significantly differentially expressed genes evaluated by limma from four comparisons: MY vs. MO, WO vs. MO, WY vs. WO, and WY vs. MY. Only genes with $|\log_2 FC| > 0.58$ ($|FC| \geq 1.5$) and FDR ≤ 0.05 are included. Column descriptions: 'logFC' represents $\log_2$-fold change of the gene between the two groups being compared; 'AveExpr' gives the average $\log_2(1+FPKM)$ for the gene across the samples used in the comparison; 't' is moderated t-statistic; 'P.Value' is the associated P-value; 'adj.P.Val' is P-value adjusted for multiple testing by the Benjamini-Hochberg method, an estimation of the false discovery rate (FDR); 'B' is the B-statistic, which is the log-odds that the gene is differentially expressed.

Supplementary Table S3. IPA canonical pathway analysis. Lists of IPA pathways significantly enriched with differentially expressed genes from MY vs. MO and WO vs. MO comparisons. Pathways with $-\log_{10}(P) \leq 1.3$ ($P \geq 0.05$) have been excluded. Column descriptions: 'z-score' indicates the prediction of activation or inactivation of the pathway, in which z-score ≥ 2 is considered significantly activated, z-score ≤ -2 is considered significantly inactivated, and z-score = NaN means IPA could not calculate the z-score; 'ratio' indicates the ratio between the number of differentially expressed genes and the number of genes in the pathway.
